# Deep Sequencing-Based Transcriptome Analysis Reveals the Regulatory Mechanism of *Bemisia tabaci* (Hemiptera: Aleyrodidae) Nymph Parasitized by *Encarsia sophia* (Hymenoptera: Aphelinidae)

**DOI:** 10.1371/journal.pone.0157684

**Published:** 2016-06-22

**Authors:** Yingying Wang, Da Xiao, Ran Wang, Fei Li, Fan Zhang, Su Wang

**Affiliations:** 1 Institute of Plant and Environment Protection, Beijing Academy of Agriculture and Forestry Sciences, Beijing, China; 2 College of Plant Protection, Nanjing Agricultural University, Nanjing, China; French National Institute for Agricultural Research (INRA), FRANCE

## Abstract

The whitefly *Bemisia tabaci* is a genetically diverse complex with multiple cryptic species, and some are the most destructive invasive pests of many ornamentals and crops worldwide. *Encarsia sophia* is an autoparasitoid wasp that demonstrated high efficiency as bio-control agent of whiteflies. However, the immune mechanism of *B*. *tabaci* parasitization by *E*. *sophia* is unknown. In order to investigate immune response of *B*. *tabaci* to *E*. *Sophia* parasitization, the transcriptome of *E*. *sophia* parasitized *B*. *tabaci* nymph was sequenced by Illumina sequencing. *De novo* assembly generated 393,063 unigenes with average length of 616 bp, in which 46,406 unigenes (15.8% of all unigenes) were successfully mapped. Parasitization by *E*. *sophia* had significant effects on the transcriptome profile of *B*. *tabaci* nymph. A total of 1482 genes were significantly differentially expressed, of which 852 genes were up-regulated and 630 genes were down-regulated. These genes were mainly involved in immune response, development, metabolism and host signaling pathways. At least 52 genes were found to be involved in the host immune response, 33 genes were involved in the development process, and 29 genes were involved in host metabolism. Taken together, the assembled and annotated transcriptome sequences provided a valuable genomic resource for further understanding the molecular mechanism of immune response of *B*. *tabaci* parasitization by *E*. *sophia*.

## Introduction

The whitefly *Bemisia tabaci* (Hemiptera: Aleyrodidae), is well known as a worldwide invasive pest and may cause severe damage to various vegetables by feeding on phloem sap and transmitting many viruses [1]. It is a complex species containing at least 30 cryptic species [2]. B and Q-types are two most economically damaging and invasive species [3]. There are many studies focus on biological characterization, resistance, invasive mechanism, and biological control of *B*. *tabaci* [4–12]. Over the past years, *B*. *tabaci* has demonstrated a remarkable resistance to many groups of chemical insecticides [13–16]. Due to the rapid resistance development, it is necessary to explore an alternative and effective management strategy to control *B*. *tabaci*. Parasitoid or parasitoid–produced regulatory molecules can be used to improve conventional pest control strategies.

Endoparasitoids have been identified as very important natural enemies of various arthropods, and could be used as biological control agents[17–19]. Hymenopteran endoparasitoids deposit their eggs into the host insect haemocoel, whose larvae feed on the host until its death [20–21]. *Encarsia sophia* is one of the specific parasitoids of *Aleyrodidae* species and has been used as efficacious classic biological control agents in many regions [22]. It can parasitize all instar nymphs of *B*. *tabaci*, especially the third and fourth instar nymphs [23]. The female wasp is generated by a bisexual process, but the male wasp is produced by autoparasitism [24]. Homogeneous *E*. *sophia* prefers to lay male eggs in the host parasitized by the heterogeneous wasp. When *E*. *sophia* and other kinds of wasps are raised or released together, the antecedent colonizers should inhibit the colonization of followers [25]. Previous studies have shown that *E*. *sophia* has strong plasticity adaption abilities[26].

However, the relationships between endoparasitoids and their hosts are complicated and involve long-term co-evolution. Many studies have investigated parasitoid biological characteristics, chemical communication, phylogenetic co-evolution, and physiological responses [27]. An increasing number of researchers have focused on revealing the physiological mechanism underlying the parasite induced immune defensive system and the biological development of hosts in order to estimate the co-evolution process between parasitoids and their hosts [28–31]. Although several reports have concentrated on the molecular regulation mechanisms, there have only been a few descriptions of related, functional genes [32,33]. Furthermore, the limitations of previous research methods has led to the development of high-throughput RNA sequencing technology (RNA-Seq)[34].

RNA-Seq is widely used to obtain transcriptomes of the organism, tissue, or organ, to identify genes that were regulated under certain conditions, and to reveal the regulatory mechanisms in different organisms [35–39]. In recent years, RNA-Seq has increasingly being applied in the biological agents to reveal the interaction mechanisms in the complex parasitoid-host system. Transcriptome profiling of organism under parasitization helps us to obtain a better understanding of host responses and effect on host’s growth, development. As a model species, *Drosophila melanogaster* and its parasitoid wasp *Asobara tabida* (Hymenoptera: Braconidae) is a well-studied system. Most genes associated with insect immunity appeared to be differentially expressed after wasp parasitized [40]. Most transcriptome studies on parasitoid-host systems have focused on Lepidoptera and Coleoptera, such as *Plutella xylostella*, *Chilo suppressalis*, *Tenebrio molitor* and *Octodonta nipae* [41–44]. A previous study showed that another parasitoid, *Eretmocerus mundus* may parasitize *B*. *tabaci* and induce the specific transcription of functional genes related to immune responses in the host [45]. However, the host manipulation by the parasitoid is species-specific, and the molecular mechanism of immune system in *B*. *tabaci* parasitization by *E*. *sophia* has not yet been explored. In this study, we used deep sequencing to explore *B*. *tabaci* response to *E*. *sophia* parasitization. Our results demonstrate that immune- and metabolic-related genes that are differentially expressed in parasitized versus non-parasitized *B*. *tabaci* nymph.

## Materials and Methods

### Insects Rearing and Parasitization

The biotype Q of *Bemesia tabaci* was obtained from the greenhouse at the Beijing Academy of Agriculture and Forestry. All experimental populations were derived from one pairs of newly emerged *B*. *tabaci* female and male. In our laboratory, the *B*. *tabaci* was reared on cotton plants (Zhong-mian-suo 49) in insect proof cages at 26 ± 1°C, and with a photoperiod of 15L: 9D. The purity of the cultures was monitored every three to five generations using the random amplified polymorphic DNA-polymerase chain reaction technique with COI gene [46]. *E*. *sophia* was obtained from the greenhouse at Beijing Academy of Agriculture and Forestry. All whitefly instar nymph stages were provided as hosts to *E*. *sophia*. Then approximate fifty *E*. *sophia* (female to male ratio of 8:1) individuals were released into cages to breed and the newly emerged female and male as parents for five generations breeding.

Thirty pairs of whiteflies were fed on cotton leaf in a micro insect cage and the fresh cotton leaf were provided every 24 hours. When they had reached later 3^rd^ or early 4^th^ instar, they were transferred in culture dish with a piece of cotton leaf, whose petiol were wrapped into soggy cotton, and then the mated *E*. *sophia* was released into *B*. *tabaci* rearing cage for parasitization. Sixty paired *E*. *sophia* were released into one culture dish. Wasp *E*. *sophia* were removed after 2 hours parasitization. The first group of samples was collected at 24-hr after parasitization (24AP). At this time period, the parasitoids were at the egg stage in which the embryo had formed and gradually began to move. The brown substance in the egg began to accumulate and chorion had appeared. In other words, the parasitoid possessed immune regulation ability, but the ability was not strong at the egg stage. Therefore, we could identify the immune defense response of the host against the parasitoid. The second sampling period was 72-hr after parasitization (72AP) when the wasps reach larval stage move around and absorb nutrition from the host. At this time, *E*. *sophia* may start to regulate host development and metabolism to finish their own development in whiteflies. Each treatment and control had three replicates.

### cDNA Library Construction and Illumina Sequencing

Total RNA was extracted from all nymph samples using TRIzol^™^ reagent (Invitrogen, Carlsbad, CA, USA) according to the manufacturer’s instruction and treated with DNaseI. The concentration and integrity of RNA sample were determined using 2100 Bioanalyzer (Agilent Technologies). The first- and second- strand cDNA synthesis, end reparation, addition of “A” bases to 3' ends, ligation of adapters at the end of DNA fragments, and PCR amplification. The cDNA library was qualified and quantified with an Agilent 2100 Bioanalyzer and ABI StepOnePlus Real-time PCR system, respectively, and then sequenced using the Illumina HiSeq^™^2000 platform at the Beijing Genomics Institute (BGI, Shenzhen, China).

### Transcriptome Analysis

In order to obtain clean reads, the low quality and adapter-polluted reads were removed from raw data. The good quality reads were assembled using Trinity[47] and assembled sequences were output as unigenes. All raw sequencing data have been deposited in NCBI Sequence Read Archive (SRA) database (http://www.ncbi.nlm.nih.gov/sra) with the following accession numbers: SRR1909644 (24AP), SRR1909651(72AP), SRR1909652 (CK-24AP), and SRR1909653 (CK-72AP). All the open reading frames (ORF) of unigene in *B*. *tabaci* were identified. If a unigene had many ORFs, we selected the longest one.

The unigenes were used for BlastX search and annotation against the NCBI non-redundant (nr) (http://blast.ncbi.nlm.nih.gov/Blast.cgi), Swiss-Prot (http://expasy.org/tools/blast), Kyoto Encyclopedia of Genes and Genome (KEGG, http://www.genome.jp/kegg/) databases with an E-value cut-off of 10^-5^. Gene Ontology (GO) annotation of unigenes was analyzed using the Blast2Go software [48], and GO functional classification for all unigens was performed using the WEGO software [49]. In the absence of *B*. *tabaci* and *E*. *sophina* genome sequences, we selected eight transcriptome datasets of *B*. *tabaci* from the NCBI database, and try to utilize the annotation that were the most closely related to *B*. *tabaci* gene in the parasitized library.

### Differentially Expressed Gene (DEG) Analysis

In order to find all the differentially expressed genes, the same FPKM (Fragments Per Kilobase per Million fragments) value of unigene was first calculated for the treatment and control groups [50]. The results were displayed as fold changes, *p*-values and q-values. According to the q-value (*p*-value’s statistical result after PFR (Positive False Rate) correction), a q-value less than 0.05 or the absolute value of fold change greater than 2 represented a significant difference between the treatment and the control.

### Quantitative Real-time PCR (qRT-PCR) Validation

The quantitative real-time PCR technique was used to verify the reliability of the deep sequencing. Nine differentially expressed genes were randomly selected. The *β-actin* gene was used for normalization. The four RNA samples represented nymphs at 24AP and 72AP, and their respective control (non-parasitized nymphs) at the same developmental stages.

First-strand cDNA was synthesized from the total RNA (1.2 μg) by using PrimeScripTM 1^st^ Strand cDNA Synthesis Kit (TaKaRa) with oligo (dT)_18_ as primer following the manufacture’s protocols. The reaction system consisted of 10 μl of SYBR Green, 0.4 μl of ROX, 2 μl of diluted cDNA, 0.4 μl of each primer and 6.8 μl of distilled water. The reactions were loaded on the CFX96^™^ Real-Time PCR Detection System (Bio-Rad, Hercules, CA) under the following conditions: 50°C for 2 min; 95°C for 2 min; and 40 cycles of 95°C for 10s, 60°C for 15s, and 72°C for 20s, followed by melting curve generation (68°C to 95°C). Data analysis was performed by one-way ANOVA following by Tukey’s test using SPSS software.

## Results and Discussion

### Illumina Sequencing and *de novo* Assembly

**Fig 1:**
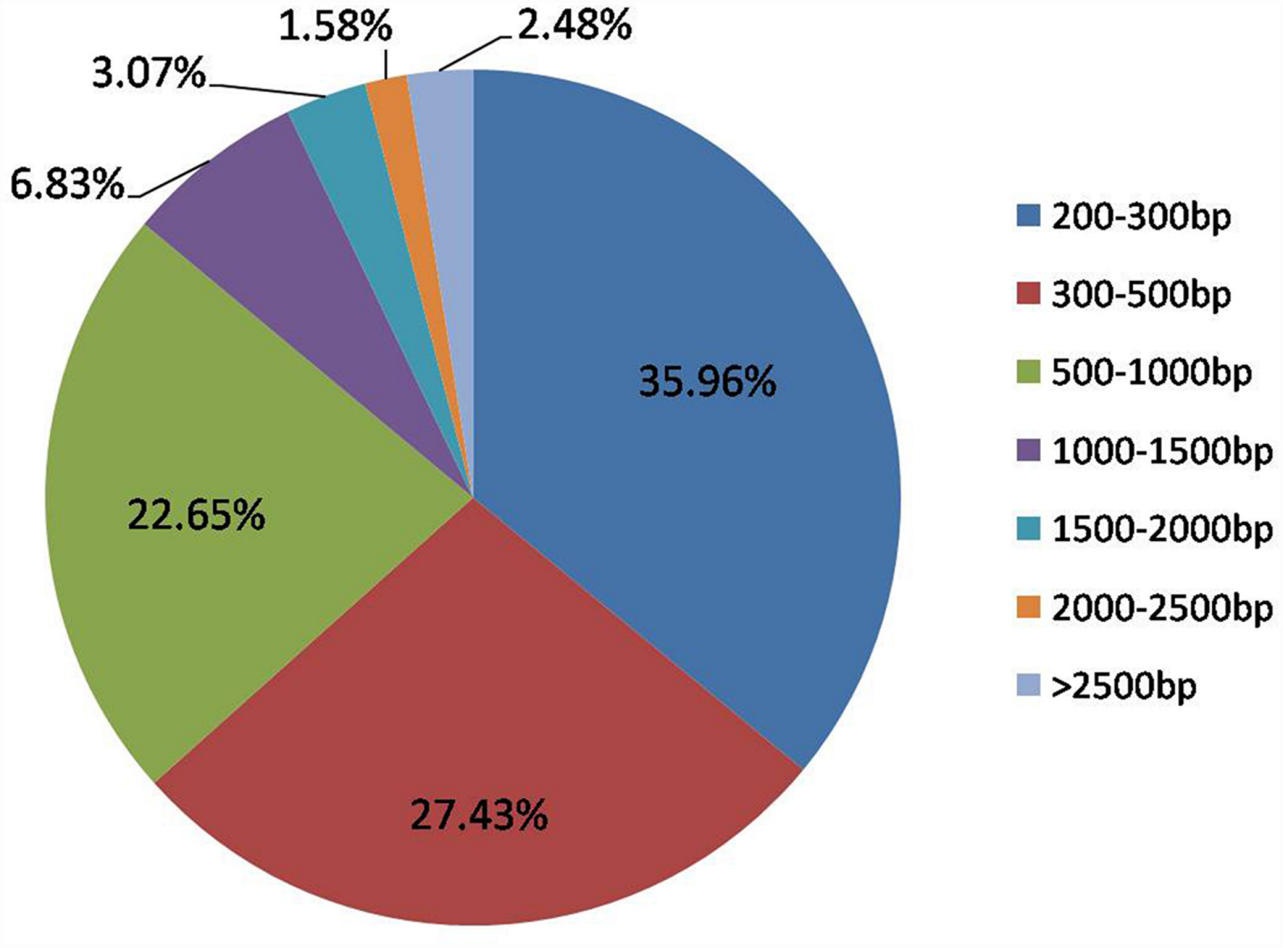
Distribution of unigene lengths in the *B*. *tabaci* transcriptome. *De novo* assembly of RNA-seq data produced 292,696 unigenes between 201–28,036bp in length.

In order to know how *E*. *sophia* parasitization regulated *B*. *tabaci* development, immune-response and the differences in regulatory mechanisms between *E*. *sophia* egg- and larvae-stages. Approximately, 35 million and 39 million reads were generated from non-parasitized and parasitized *B*. *tabaci* nymphs at 24AP, respectively, and 31million and 37 million reads were from non-parasitized and parasitized *B*. *tabaci* nymphs at 72AP, respectively. *De novo* assembly produced 292,696 *B*. *tabaci* unigenes with an average size of 616 bp. Of these unigenes, 35.96% were between 200 and 300bp, 27.43% were between 300 and 500bp, 22.65% were between 500 and 1000bp and 13.96% had nucleotide lengths above 1000bp (Fig 1).

### Functional Annotation and Classification

For functional annotation, the 292,696 unigenes were aligned to the GenBank protein databases with a cut-off E-value of 10^−5^ using BLASTx. Using this approach, 46,406 unigenes (15.8% of all unigenes) were successfully mapped. In order to predicate protein function, the unigenes were further given a gene ontology (GO) classification and subjected to KEGG pathway analysis. A total of 35,688 unigenes were annotated and assigned to GO terms, which consisted of three main categories: biological process, cellular component and molecular function. A total of 11,993 unigenes were categorized as cellular components, 12,102 unigenes were grouped under the molecular function, and 11,593 unigenes under biological processes. KEGG pathway analysis indicated that there were 4,721 unigenes assigned to different pathways in which translation, signal transduction, neurodegenerative diseases, infectious diseases, and endocrine system were the main *B*. *tabaci* pathways after *E*. *sophia* parasitizzation.

### Enrichment Analysis of DEGs

**Fig 2:**
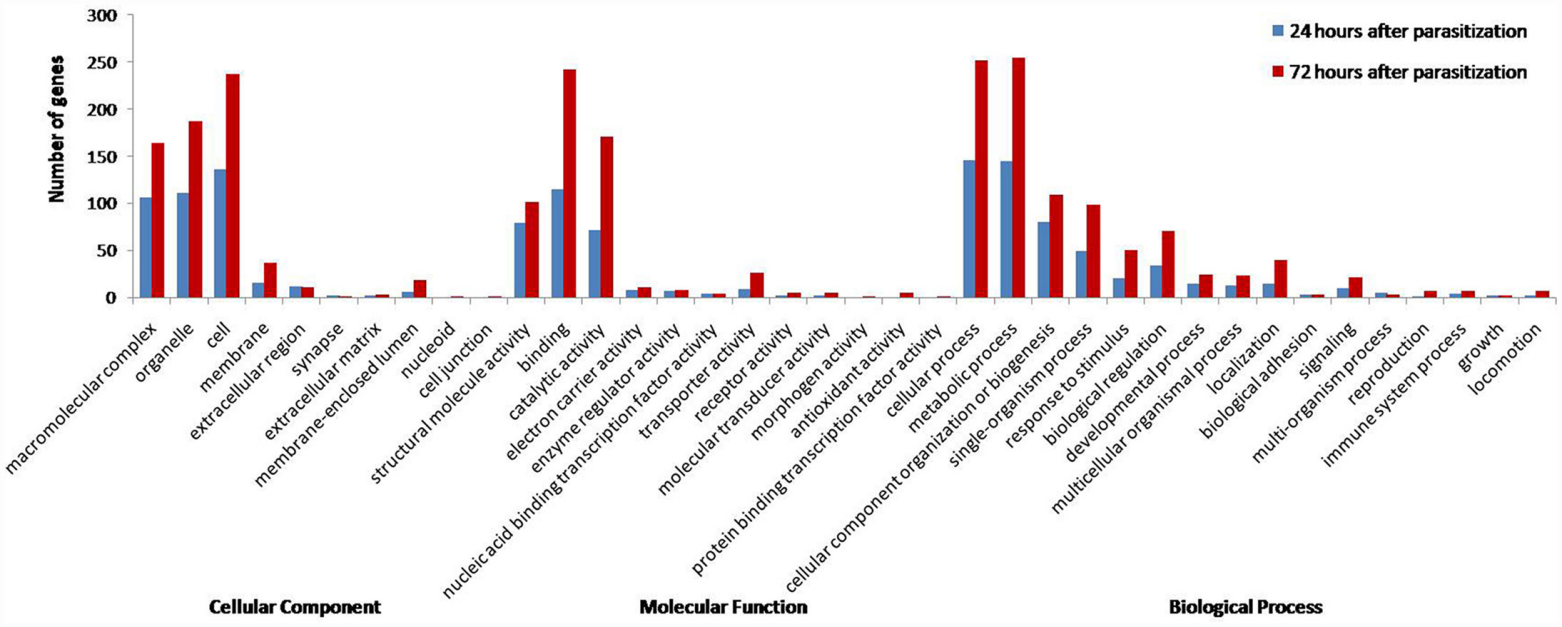
General information about genes that were differentially expressed in response to parasitization. The left figure shows the numbers of genes that were up-regulated and down-regulated at 24AP and 72AP. The right figure shows distribution of up-regulated (blue bars) and down-regulated (red bars) genes based on their fold change.

A total of 1,482 genes appeared to be significantly differentially expressed in the parasitized and non-parasitized *B*. *tabaci*, of which 852 genes were differentially up-regulated and 630 genes were differentially down-regulated (Fig 2A). At 24AP, there were 584 genes differentially expressed, of which 356 genes were up-regulated and 228 genes were down-regulated. At 72AP, there were 1,270 genes differentially expressed, of which 698 genes were up-regulated and 572 genes were down-regulated. Out of all of regulated genes, 202 up- and 170 down-regulated genes were found at both time points (Fig 2B) and more genes were up-regulated than that of the down-regulated genes at both 24AP and 72AP (Fig 2A). Furthermore, there was a significant difference in the numbers of differentially expressed genes at 24 hours than at 72 hours after parasitization. When *E*. *sophia* emerge in the larvae stage, more genes seemed to be involved in regulatory responses as compared to the egg stage. During the larvae stage, the parasitoid could move freely and began to feed on the host tissues. The distribution of the regulated genes indicated that their expression levels (>95%) were between two- to six-fold higher than at the egg stage (24 AP). Only a few genes changed more than six-fold (Fig 2).

**Fig 3:**
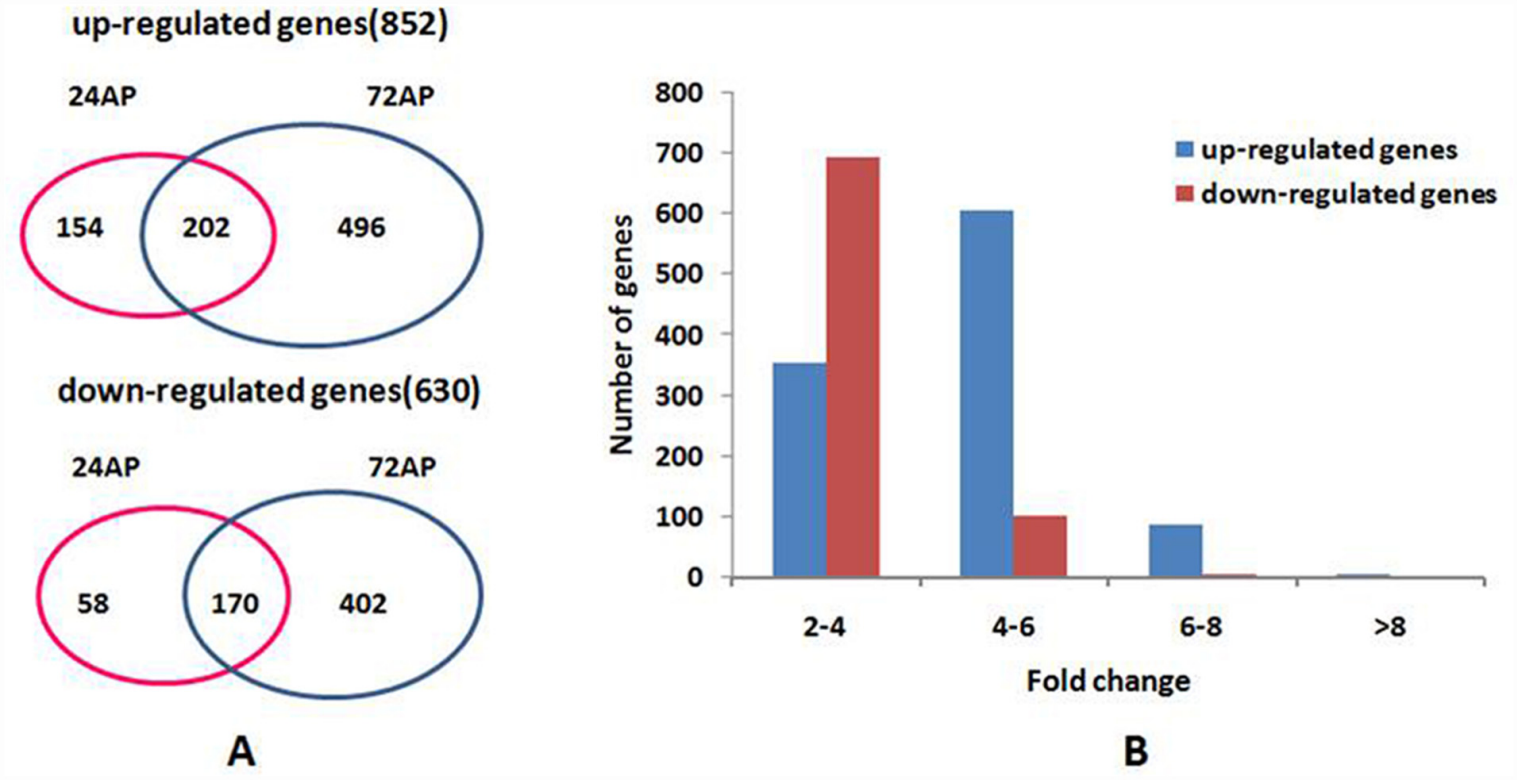
GO annotation of differentially expressed genes at 24APand 72AP (level 2). At 24AP, the program categorized 390 unigenes in the cellular component category, 297 unigenes in the molecular function category, and 542 unigenes in the biological process category. At 72AP, the program categorized 732 unigenes in the cellular component category, 580 unigenes in the molecular function category, and 971 unigenes in the biological process category.

GO analysis revealed that the DEGs were mainly categorized in the cellular component cluster, that focus on macromolecular, organelle, and cellular levels. In the molecular function cluster, the DEGs were mainly found in structural molecule, binding, and catalytic activity. In the biological process cluster, the DEGs were mainly categorized in cellular and metabolic processes, and cellular component organization or biogenesis (Fig 3). In addition, more genes were involved in cellular processes, metabolic processes, single-organism processes, response to stimuli, biological regulation, localization, and cellular component organization or biogenesis at 72AP. Translation and signal transduction were the two most important pathways according to the KEGG pathways analysis. For KEGG enrichment analysis, genes involved in the immune system, nervous system, endocrine system, and metabolic activities were differentially expressed. The above results showed that parasitization had a great impact on the normal life activities of the host.

### Effects of Parasitism on the Transcription of Host Immune-related Genes

Vertebrates have a set of immune defense mechanisms that include innate immunity and adaptive immunity, but invertebrates only have innate immunity protection [51]. Insects will initiate their innate immune response when encounting foreign agents, such as bacteria, fungi, virus, and parasitoid. The immune system of insects can be divided into two categories: 1) humoral defense, including the antimicrobial peptides, reactive intermediates of oxygen, melanin formation and clotting; and 2) cellular defense mainly based on haemocytes, such as phagocytosis, encapsulation, microaggregation and nodulation [52–54]. Two defense mechanisms are associated with a wide range of immune-related genes.

Our sequencing results indicated that *E*. *sophia* parasitism had a significant impact on the transcription of immune-related genes in *B*. *Tabaci* nymph (Table 1). We identified several up-regulated genes with homologs known to be involved in immune responses in insects, such as: *defensin*, *knottin*, *serpin I2*, *laminin*, *spectrin*, and *apolipophorin*. Defensin is an antimicrobial peptide, which acts as an innate immunity effector molecule and provides the first protection from pathogen infection. After parasitization by *E*. *sophia* at 24AP, we found that the transcription of *defensin* was up-regulated in *B*. *tabaci* nymph. Our results were consistent with previous studies that the mRNA levels of defensin in *D*. *melanogaster* and *Phlebotomus duboscqi* were significantly increased after parasitization [55, 56]. Although the main action targets of *defensin* are bacteria and fungi, it also plays a role in the host-parasitoid system. Knottins are mini proteins that are present in many different organisms and have various biological functions [57]. After parasitization by *E*. *sophia* at both 24AP and 72AP, four *knottins* were over-expressed. Like defensin, it is also an important antimicrobial peptide.

**Table 1:** Immune-related genes differentially expressed in *B*. *tabaci* after being parasitized by *E*. *sophia*.

Gene ID	Length	Gene name	NP-FPKM	P-FPKM	Fold change	*P*-value	q-value
**Genes up-regulated at 24AP**							
Unigene_131748	1458	Spectrin alpha chain (*Drosophila melanogaster*)	0.131	1.893	3.85	2.77E-05	0.0163
Unigene_111268	648	Probable chitinase 3 (*Drosophila melanogaster*)	0.211	4.937	4.55	1.18E-05	0.00865
Unigene_145476	225	Defensin (*Galleria mellonella*)	0.795	10.671	3.75	0.000119	0.0489
Unigene_183100	744	Hemocyanin (*Palinurus vulgaris*)	0.421	5.305	3.66	3.50E-05	0.0195
Unigene_190217	2130	Protein toll (*Drosophila melanogaster)*	0.0154	1.793	6.86	5.15E-06	0.00455
Unigene_194849	909	Apolipophorins (*Locusta migratoria*)	0.264	4.275	4.02	4.63E-06	0.00417
Unigene_155504	573	Protein disulfide-isomerase (*Drosophila melanogaster*)	0.189	4.503	4.57	1.20E-08	4.20E-05
Unigene_83201	1410	Cytospin-A (*Takifugu rubripes)*	0.0439	1.013	4.53	0.000115	0.0476
Unigene_154109	477	Apoptosis 2 inhibitor (*Drosophila melanogaster*)	0.0845	11.267	7.06	2.71E-06	0.00275
Unigene_134383	2097	Zinc finger MIZ domain-containing protein 1 (*Homo sapiens*)	0.112	1.585	4.93	2.40E-05	0.0148
Unigene_329886	2853	Ankyrin-3 (*Homo sapiens*)	3.389	22.543	2.73	1.69E-05	0.0114
Unigene_268316	1131	Cytochrome P450 6a2 (*Drosophila melanogaster*)	3.265	26.698	3.03	1.66E-06	0.00190
Unigene_288430	1278	Cytochrome P450 6k1 (*Blattella germanica*)	1.451	60.115	5.37	1.78E-15	1.46E-10
Unigene_277195	249	*Bemisia tabaci* putative antimicrobial knottin protein Btk-4 (*Bemisia tabaci*)	7.0717	113.992	4.01	1.14E-09	6.57E-06
**Genes up-regulated in the 72AP**							
Unigene_154832	372	Arginine kinase (*Apis mellifera*)	0.349	2.993	3.10	3.48E-05	0.0194
Unigene_156269	633	Serpin I2 (*Mus musculus*)	0.0552	2.228	5.33	9.73E-05	0.0419
Unigene_330609	1089	Serine protease homolog 42 isoform 2 (*Nasonia vitripennis*)	0.0441	3.424	6.28	3.75E-06	0.00353
Unigene_136705	681	GILT-like protein C02D5.2 *Caenorhabditis elegans* (*Caenorhabditis elegans*)	0.1009	8.852	6.45	1.94E-06	0.00214
Unigene_55874	699	cAMP-dependent protein kinase catalytic subunit (*Drosophila melanogaster*)	0.1532	3.324	4.44	4.84E-05	0.0249
Unigene_126734	516	guanine nucleotide-binding protein G (q) subunit alpha (*Homarus americanus*)	0.112	2.325	4.37	7.49E-06	0.00607
Unigene_70023	1074	Alpha-actinin, sarcomeric (*Drosophila melanogaster*)	0.191	2.429	3.67	6.57E-05	0.0314
Unigene_116220	753	Apoptosis inhibitor 5 (*Homo sapiens*)	0.0541	2.220	5.36	9.03E-05	0.0396
Unigene_116141	804	Casein kinase II subunit alpha (*Spodoptera frugiperda*)	0.281	3.880	3.78	3.64E-05	0.0201
Unigene_231151	894	Serine/threonine-protein kinase PAK 1-like isoform 1 (*Bombus impatiens*)	0.0328	1.632	5.63	3.64E-05	0.0209
Unigene_213155	1212	Cytochrome P450 4e3 (*Drosophila melanogaster*)	0.327	3.011	3.20	6.21E-05	0.0302
Unigene_214185	1041	Probable cytochrome P450 6a18 (*Drosophila melanogaster*)	0.491	4.686	3.25	1.69E-05	0.0114
Unigene_161936	1362	Laminin subunit beta-1 (*Drosophila melanogaster*)	0.131	2.682	4.35	6.93E-05	0.0326
Unigene_232028	873	Cytochrome P450 4g15 (*Drosophila melanogaster*)	1.948	12.021	2.63	7.07E-05	0.0331
Unigene_239924	948	Cytochrome P450 4C1(*Blaberus discoidalis*)	0.469	4.649	3.31	2.31E-05	0.0143
Unigene_288430	1278	Cytochrome P450 6k1 (*Blattella germanica*)	0.919	6.934	2.91	1.50E-05	0.0103
**Genes up-regulated in the 24AP and 72AP**							
Unigene_143480	303	*Bemisia tabaci* putative antimicrobial knottin protein Btk-1 (*Bemisia tabaci*)	16.101	283.469	4.14	1.86E-10	1.64E-06
			15.014	127.85	3.09	9.91E-07	0.00126
Unigene_244685	195	*Bemisia tabaci* putative antimicrobial knottin protein Btk-2 (*Bemisia tabaci*)	33.883	419.491	3.63	5.82E-08	0.000142
			21.196	226.199	3.42	1.19E-07	0.000247
Unigene_138814	183	*Bemisia tabaci* putative antimicrobial knottin protein Btk-3 (*Bemisia tabaci*)	7.869	288.573	5.20	8.44E-15	5.79E-10
			6.807	113.886	4.06	5.77E-10	3.94E-06
Unigene_158224	552	Ras-like protein 3 (*Drosophila melanogaster*)	0.138	1.695	3.62	1.30E-05	0.00929
			0.131	3.582	4.78	7.97E-09	3.13E-05
Unigene_74024	576	Ras-like GTP-binding protein Rho1 (*Drosophila melanogaster*)	0.119	5.015	5.40	3.71E-06	0.00326
			0.379	8.120	4.42	3.02E-07	0.000506
Unigene_45734	2199	Serine/threonine-protein kinase SRPK3 (*Bombus impatiens*)	0.0793	0.907	3.61	9.18E-05	0.0402
			0.0602	2.0787	5.11	1.25E-07	0.000255
Unigene_154175	711	Heat shock 70 kDa protein cognate 3 (*Drosophila melanogaster*)	0.501	5.758	3.52	7.29E-07	0.000997
			0.0908	5.741	5.98	4.75E-10	3.37E-06
Unigene_244154	591	Actin-5C (*Anopheles gambiae*)	2.036	31.216	3.94	8.39E-08	0.000189
			2.459	20.452	3.06	2.43E-05	0.0149
Unigene_134315	663	Casein kinase II subunit beta (*Rattus norvegicus*)	0.112	1.585	3.82	8.55E-05	0.0381
			0.0713	2.142	4.91	6.10E-06	0.00519
Unigene_47109	912	Guanine nucleotide-binding protein subunit beta-like protein (*Drosophila melanogaster*)	5.457	31.448	2.53	5.61E-05	0.0278
			3.347	66.897	4.32	3.95E-11	4.93E-07
Unigene_185113	1365	Glycogen synthase kinase 3 beta (*Nasonia vitripennis*)	0.0137	0.797	5.85	0.000112	0.0466
			0.0174	0.915	5.71	2.70E-05	0.0165
Unigene_218239	321	Cofilin (*Drosophila melanogaster*)	0.774	8.850	3.51	4.06E-05	0.0218
			1.279	14.450	3.50	7.13E-06	0.00584
**Genes down-regulated in the 72AP**							
Unigene_201980	897	Paramyosin, short form (*Drosophila melanogaster*)	143.013	21.903	-2.71	3.04E-05	0.0176
Unigene_201985	195	Paramyosin, long form (*Drosophila melanogaster*)	41.061	6.947	-2.56	9.20E-05	0.0402
Unigene_201725	1752	Chorion peroxidase (*Drosophila melanogaster*)	2.286	0.216	-3.40	1.20E-05	0.00878
Unigene_176466	1101	Cathepsin B (*Mus musculus*)	28.719	3.253	-3.14	6.99E-07	0.000964
Unigene_274030	624	Superoxide dismutase [Cu-Zn](SODC) (*Drosophila willistoni*)	2.831	0.180	-3.97	3.74E-06	0.00352
Unigene_286469	2118	Peroxidase (PERO) (*Drosophila melanogaster*)	32.555	3.919	-3.05	1.45E-06	0.00171
Unigene_224486	1527	Catalase (CATA) (*Riptortus pedestris*)	21.707	3.362	-2.69	1.70E-05	0.0114
Unigene_192238	462	Troponin C, isoform 1 (*Drosophila melanogaster*)	58.280	5.885	-3.31	1.79E-07	0.000336
Unigene_253831	1491	Probable cytochrome P450 303a1 (*Drosophila melanogaster*)	34.031	4.682	-2.86	6.32E-06	0.00534

**Fig 4:**
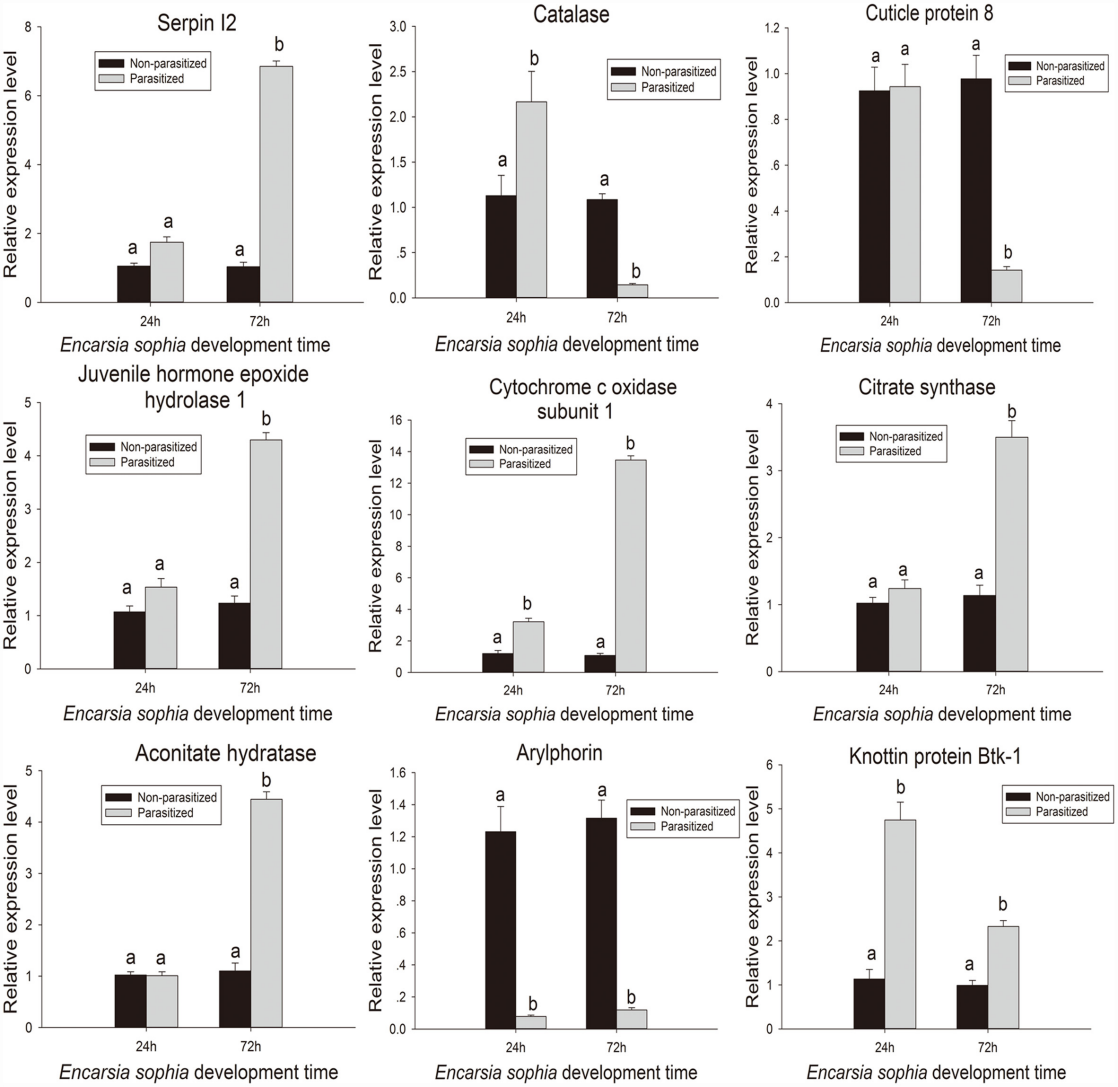
A qRT-PCR analysis of nine randomly selected genes from *B*.*tabaci* that showed relative expressions at 24AP and 72AP. Black stands for the control and gray stands for a parasitized sample. The expression levels of the controls were regarded as 1. Error bars indicate standard deviations of the average from three replicates. The same letter above the error bar means that there was no significant difference at the 0.05 level by Duncan’s test.

*Serpin I2* was one of the genes having higher levels of up-regulation (5.33-fold) at 72AP in RNA-seq analysis. Quantitative RT-PCR analysis (Fig 4) also show that it was up-regulated by 6.85 folds. Serine proteases are important immune regulatory proteins which play a significant role in the activation of the prophenoloxidase (PPO) cascade. The cascade activation eventually causes melanization to kill parasitized wasp through choking [58], however, serine protease inhibitor (serpin) can prevent the serine proteases activated melanization and weaken host defense for wasp parasization. Although studies have shown that serpins can be regulated by the parasitoids infestation in many hosts, their transcriptional levels are different in different parasitoid-host systems, and even in the same parasitoid-host system, two opposite situations may occur. Mahadav *et al*. and Song *et al*. found that serpins were down-regulated in parasitized *B*. *tabaci* nymphs and *P*. *xylostella* larvae [26,59], while Etebari *et al*.[41] discovered that serpins were up-regulated 2- to 7-fold after *P*. *xylostella* parasitization by *Diadegma semiclausum*. In *C*. *chilonis* parasitized *C*. *suppressalis*, three up-regulated and three down-regulated serpins were identified in the fatbody [42]. Different serpins may play different roles in immune defense.

Cellular immunity is another important component of the insect immune system. Laminin can stimulate cell adhesion and cell movement. Cofilin is an actin-binding protein which promotes cell migration and movement by changing the adhesion between cells and the extracellular matrix. Actin plays a significant role in facilitating cellular activities. The up-regulation of these genes showed that the host enhanced hemocyte encapsulation by reinforcing the extension and adhesion of hemocytes. *laminin*, *cofilin*, and *actin* were identified in our study and they were over-expressed at 24AP and 72AP. *Ras3* and *Rho1* are related to cellular immunity in *D*. *melanogaster* [40,60]. In our study, two genes were also identified significantly differentially expressed after parasization which may also be involved in the immune response of *B*. *tabaci*. At 24AP and 72AP, these genes were consistently over-expressed, which indicated that the cellular immunity not only defend parasitoid embryo and larval attacking.

Superoxide dismutase (SOD), peroxidase (POD) and catalase (CAT) are three common enzymes in organisms. Organisms produce reactive oxygen species (ROS) under environmental stresses, which is cytotoxic to cells. However, the organism utilizes these protective enzymes to eliminate redundant ROS and protect themselves from damage [61]. When *Trichoplusia ni* is infected by baculoviruses, the expression of manganese superoxide dismutase (MnSOD) significantly reduces oxidative damage [62]. Zhu *et al*. also discovered that the transcriptional levels of *Tenebrio molitor* superoxide dismutases were up-regulated following bacterial infection or parasitization by *Scleroderma guani* [63]. In our study, the expression of *SODC*, *PERO*, and *CATA* were suppressed two-to four-fold at 72AP, but did not show significant changes at 24AP. At 72AP, the *E*. *sophia* reaches the larval stage and the damage to host becomes worse than that of egg stage. The decrease in the transcription of protective enzymes showed the parasitoid immune suppressive strategy.

In our study, some genes involved in insecticide resistance or detoxification were found to be differentially expressed under parasitization. Although these genes have no direct connection with defense against parasitoids attacking, they can be regarded as a stress response caused by parasitoid secretions. Takeda *et al*.[64] confirmed that the activities of glutathione-S-transferase (GST) and cytochrome P450 (CYP) increased in parasitized *P*. *xylostella* larvae. We also found most of the cytochrome P450 genes were highly expressed after being parasitized by *E*. *sophia*. Some genes were over-expressed at 24AP and others were over-expressed at 72AP.

Heat shock proteins (HSPs) are recognized as a family of highly conserved chaperones which respond to all kinds of environmental stress factors, such as heat, toxins, UV radiation, and invading pathogens by protecting protein from misfolding and denaturation [65]. We identified three heat shock protein genes, which were homologous with *D*. *melanogaster* and *Anopheles albimanus*, and are involved in *B*. *tabaci* development. In addition, heat shock 70 kDa protein cognate 3 was found to participate in the immune response. Therefore, we deduced that heat shock protein families can defend the host from damage by participating in the immune response and *B*. *tabaci* development.

### Effects of parasitism on the transcription of host development-related genes

The parasitoids complete their development by absorbing the host’s hemolymph and tissues. However, the development of the parasitoid and the host are synchronous. A previous study found that *Aphidiu servi* parasitized *Acyrthosiphon pisum* late-stage nymph stopped growth [30]. *B*. *tabaci* late nymphs parasitized by *Encarsia bimaculata* also stop growing [66]. After *Encarsia formosa* parasitized *Trialeurodes vaporariorum* Westwood nymph, the wasp didn’t molt to until host nymph reached to last instar [67]. In order to complete development, the parasitoids have to change the host’s development to match their own growth. In some cases, parasitoids suppress host’s development and accelerate the host’s early-maturity [68], while, other parasitoids prolong host’s development to meet their own developmental needs. Previous studies have proposed that the wasp might control host’s development through regulating the juvenile hormone and ecdysone levels [69,70].

Juvenile hormone epoxide hydrolases (JHEHs) have been identified as regulatory proteins in the catabolism of juvenile hormones [71,72]. A previous study showed that JHEH transcript levels were down-regulated more than two-fold in *P*. *xylostella* after parasitization by *D*. *semiclausum* [41]. However, Wu *et al*. [42] discovered that JHEH and juvenile hormone esterase (JHE) transcript levels increased in *C*. *suppressalis* after *C*. *chiilonis* parasitization. Based on our transcriptome data, parasitization by *E*. *sophia* led to *JHEH1* up-regulation at 72AP (Table 2 & Fig 4). However, up-regulation of larvae cuticle protein and down-regulation of pupal cuticle protein might imply that the parasitoid suppressed the host’s development. Thus, the high concentrations of JH may lead to up-regulation of JHEHs and their activity in order to maintain the balance.

**Table 2:** Developmental-related genes differentially expressed in *B*. *tabaci* after being parasitized by *E*. *sophia*.

Gene ID	Length	Gene name	NP-FPKM	P-FPKM	Fold change	*P*-value	q-value
**Genes up-regulated in the 24AP**							
Unigene_119370	660	RNA-binding protein squid (*Drosophila melanogaster*)	0.0369	2.346	5.99	7.62E-05	0.0350
Unigene_137439	1092	Protein slit (*Drosophila melanogaster*)	0.0214	1.340	5.97	8.09E-05	0.0365
Unigene_122721	588	Hormone receptor 4 (*Drosophila melanogaster*)	0.218	3.772	4.12	8.24E-05	0.0370
Unigene_58772	369	Fatty acid-binding protein 3, muscle and heart (*Camponotus floridanus*)	1.091	18.331	4.07	2.60E-05	0.0157
**Genes up-regulated in the 72AP**							
Unigene_47461	1074	Plexin-B (*Drosophila melanogaster*)	0.071	1.835	4.69	1.65E-05	0.0111
Unigene_163674	741	Larval cuticle protein A3A (*Tenebrio molitor*)	0.095	10.464	6.77	2.50E-10	2.07E-06
Unigene_300307	327	Larval cuticle protein 8 (*Drosophila melanogaster*)	0.209	96.444	8.85	5.56E-11	6.40E-07
Unigene_96322	1440	Juvenile hormone epoxide hydrolase 1(*Ctenocephalides felis*)	0.079	2.362	4.89	4.46E-07	0.000687
Unigene_55874	699	cAMP-dependent protein kinase Catalytic subunit (*Drosophila melanogaster*)	0.153	3.324	4.44	4.84E-05	0.0249
Unigene_330433	2163	Heat shock protein 83 (*Drosophila melanogaster*)	0.045	17.839	8.62	1.11E-15	1.08E-10
Unigene_240353	2013	Heat shock protein 70 B2 (*Anopheles albimanus*)	0.022	0.903	5.34	9.45E-05	0.0410
Unigene_126734	516	Guanine nucleotide-binding protein G (q) subunit alpha (*Homarus americanus*)	0.112	2.32	4.37	7.49E-06	0.00607
Unigene_156339	354	Eukaryotic translation initiation factor 4E binding protein 1(*Nasonia vitripennis*)	0.232	5.202	4.49	7.39E-07	0.001016
Unigene_157748	2775	Mediator of RNA polymerase II transcription subunit 13 (*Nasonia vitripennis*)	0.033	0.919	4.80	1.00E-05	0.00763
**Genes up-regulated at 24AP and 72AP**							
Unigene_74024	576	Ras-like GTP-binding protein Rho1(*Drosophila melanogaster*)	0.119	5.016	5.40	3.37E-06	0.00326
			0.379	8.120	4.42	3.02E-07	0.000506
Unigene_185113	1365	Glycogen synthase kinase 3 beta (*Nasonia vitripennis*)	0.0138	0.797	5.85	0.000112	0.0466
			0.017	0.915	5.71	2.79E-05	0.0165
Unigene_218239	321	Cofilin (*Drosophila melanogaster*)	0.774	8.851	3.51	4.06E-05	0.0218
			1.279	14.450	3.50	7.13E-06	0.00584
Unigene_45734	2199	Serine/threonine-protein kinase SRPK3 (*Bombus impatiens*)	0.079	0.967	3.6	9.18E-05	0.0402
			0.060	2.078	5.11	1.25E-07	0.000255
Unigene_244154	738	Small subunit ribosomal protein S6e (*Manduca sexta*)	2.036	31.21	3.07	8.39E-08	0.000189
			2.459	20.452	4.48	2.43E-05	0.0149
Unigene_131412	1218	Endoplasmin (*Nasonia vitripennis*)	0.037	0.941	4.67	6.78E-05	0.0322
			0.023	0.968	5.37	8.78E-05	0.0388
Unigene_148396	1641	Elongation factor 2 (*Drosophila melanogaster*)	2.793	23.783	3.98	9.28E-05	0.0405
			2.001	26.905	3.75	7.49E-06	0.00607
Unigene_287521	561	Myosin light chain 6 (*Apis mellifera*)	0.996	7.218	2.86	6.01E-05	0.0294
			1.267	15.772	3.64	1.23E-07	0.000253
Unigene_244154	591	Actin-5C (*Anopheles gambiae*)	2.036	31.216	3.94	8.39E-08	0.000189
			2.459	24.453	3.06	2.43E-05	0.0149
Unigene_154175	711	Heat shock 70 kDa protein cognate 3 (*Drosophila melanogaster*)	0.502	6.758	3.52	7.29E-07	0.000997
			0.908	5.741	5.98	4.75E-10	3.37E-06
**Genes down-regulated at 72AP**							
Unigene_231672	372	Adult-specific cuticular protein ACP-20 (*Tenebrio molitor*)	6.293	0.076	-3.22	0.000108	0.0453
Unigene_225094	939	Opsin-2 (*Schistocerca gregaria*)	14.373	2.031	-2.82	7.56E-06	0.00612
Unigene_192238	462	Troponin C, isoform 1/ calmodulin (*Drosophila melanogaster*)	58.280	5.885	-3.31	1.79E-07	0.000336
Unigene_196111	3882	Fatty acid synthase (*Gallus gallus*)	0.961	0.067	-3.84	8.25E-06	0.00652
**Genes down-regulated at 24AP and 72AP**							
Unigene_178448	2010	Arylphorin subunit alpha (*Manduca sexta*)	2.532	0.255	-3.31	1.13E-05	0.00838
			4.377	0.555	-2.98	1.29E-05	0.00923
Unigene_198800	390	Pupal cuticle protein Edg-84A (*Drosophila melanogaster*)	139.029	21.277	-2.71	8.31E-05	0.0372
			169.474	23.808	-2.83	5.56E-05	0.0277
Unigene_196053	447	Cuticle protein 8 (*Blaberus craniifer*)	789.210	95.603	-3.54	7.40E-05	0.0343
			1674.11	101.192	-2.84	2.30E-06	0.00245
Unigene_257893	714	Cuticle protein 7 (*Locusta migratoria*)	436.781	50.575	-3.11	0.000119	0.0487
			683.999	45.784	-3.90	5.98E-06	0.00511
Unigene_203192	468	Cuticle protein 19 (*Locusta migratoria*)	13.851	2.438	-2.51	6.23E-05	0.0302
			19.539	3.361	-2.54	4.69E-05	0.0243

### Effects of parasitism on the transcription of host metabolism-related genes

Stearoyl-CoA desaturase (SCD) is an endoplasmic reticulum enzyme that catalyzes the biosynthesis of monounsaturated FA from saturated FA [73]. SCD inactivation causes obesity and abnormal lipid metabolism and one SCD activity, *SCD1*, was induced by insulin, but inhibited by leptin [74]. We found that at 24AP and 72AP, the transcript levels of *SCD* in *B*. *tabaci* nymph were up-regulated 6.69 and 4.52 times, respectively (Table 3). Furthermore, genes involved in the insulin signaling pathway were also significantly up-regulated. Our result implied that the wasp regulated the lipid metabolism of the host in order to get more nutrients available in host and meet their own needs. A report showed that the wasp preferred to parasitize late instar larvae because of adequate nutrition [75]. Stearoyl-CoA desaturase is an essential enzyme for the parasitic *Trypanosoma brucei*, and RNA interference of *SCD* caused a reduction of the parasitemia and an increase in host survival [76]. Environmental stress can influence the organism’s metabolism, same as parasitoid infestation, which is energetically consumption process [77]. We found a high number of differentially expressed transcripts were related to organism metabolism. Metabolic changes occurred at both time points, but a greater amount and different kinds of genes were affected at 72APthan at24AP.

**Table 3:** Metabolism-related genes differentially expressed in *B*. *tabaci* after being parasitized by *E*. *sophia*.

Gene ID	Length	Gene name	NP-FPKM	P-FPKM	Fold change	*P*-value	q-value
**Genes up-regulated at 24 AP**							
Unigene_108039	489	V-type H+-transporting ATPase 16kDa proteolipid subunit (*Drosophila melanogaster*)	0.0841	4.129	5.62	1.25E-06	0.00151
Unigene_71375	777	Glutamine synthetase 2, isoform B (*Drosophila melanogaster*)	0.0445	2.586	5.86	0.00011	0.0460
Unigene_111268	900	Probable chitinase 3 (*Drosophila melanogaster*)	0.211	4.937	4.11	1.18E-05	0.00865
Unigene_316078	861	Stearoyl-CoA desaturase (*Trichoplusia ni*)	0.0307	3.181	6.69	8.97E-06	0.00697
Unigene_151198	1947	Chitin synthase A (*Spodoptera exiqua*)	0.028	1.216	5.40	3.30E-06	0.00321
Unigene_130384	1677	Fatty acyl-CoA reductase (*Drosophila melanogaster*)	0.039	1.574	5.33	4.43E-06	0.00402
**Genes up-regulated at 72 AP**							
Unigene_106282	291	Cytochrome c oxidase subunit 2 (*Nasonia qiraulti*)	0.703	14.135	4.25	8.74E-07	0.00113
Unigene_47076	450	Cytochrome c oxidase subunit 5a (*Nasonia vitripennis*)	0.208	9.349	5.50	5.53E-05	0.0276
Unigene_77855	936	F-type H+-transporting ATPase subunit beta (*Drosophila melanogaster*)	1.119	10.000	3.16	6.67E-06	0.00554
Unigene_142444	822	F-type H+-transporting ATPase subunit gamma (*Drosophila melanogaster*)	0.079	3.912	5.62	3.80E-05	0.0208
Unigene_115206	333	F-type H+-transporting ATPase subunit a (*Aedes aegypti*)	0.829	31.105	5.23	1.01E-09	6.04E-06
Unigene_72168	321	F-type H+-transporting ATPase subunit f (*Drosophila melanogaster*)	0.539	10.619	4.30	1.07E-05	0.00803
Unigene_110914	996	Glyceraldehyde 3-phosphate dehydrogenase (*Drosophila pseudoobscura*)	0.679	15.247	4.49	1.09E-10	1.07E-10
Unigene_261092	783	Citrate synthase (*Aedes aegypti*)	0.214	5.028	4.55	7.02E-09	2.83E-05
Unigene_134666	1512	Aconitate hydratase (*Nasonia vitripennis*)	0.066	1.287	4.27	9.84E-05	0.0423
Unigene_330649	972	Succinyl-CoA synthetase alpha subunit (*Drosophila melanogaster*)	0.083	2.182	4.71	1.54E-05	0.0106
Unigene_110915	786	Arylformamidase (*Cerapachys biroi*)	0.262	7.736	4.88	4.63E-07	0.000765
Unigene_110492	165	F-type H+-transporting ATPase subunit alpha (*Drosophila melanogaster*)	0.210	32.159	6.72	6.94E-07	0.000961
Unigene_239710	1395	Facilitated trehalose transporter Tret1(*Culex quinquefasciatus*)	0.352	2.810	3.00	1.41E-05	0.0410
**Genes up-regulated at 24 AP and 72 AP**							
Unigene_120237	261	Ubiquinol-cytochrome c reductase cytochrome b subunit (*Philotrypesis pilosa*)	1.147	10.895	3.25	2.39E-06	0.00251
			1.198	17.491	3.87	1.59E-08	5.24E-05
Unigene_153762	150	Cytochrome c oxidase subunit 1 (*Locusta migratoria*)	0.865	9.243	3.42	2.53E-06	0.00262
			0.856	16.724	4.29	3.12E-09	1.47E-05
Unigene_228753	570	Maltase 1 (*Drosophila virilis*)	0.340	5.047	3.89	9.82E-06	0.00748
			0.145	4.129	4.83	8.59E-06	0.00673
Unigene_228756	1071	Maltase 2 (*Drosophila virilis*)	5.034	39.472	2.97	2.39E-06	0.00251
			3.573	45.787	3.68	9.93E-09	3.70E-05
**Genes down-regulated at 24 AP**							
Unigene_241514	1599	Pyruvate kinase (*Drosophila melanogaster*)	25.087	4.04	-2.63	3.09E-05	0.0178
**Genes down-regulated at 72 AP**							
Unigene_269576	432	ATP synthase lipid-binding protein, mitochondrial (*Manduca sexta*)	66.809	10.812	-2.63	3.45E-05	0.0193
Unigene_249063	1092	Fructose-bisphosphate aldolase, class I (*Drosophila melanogaster*)	7.807	0.927	-3.07	5.59E-06	0.00484
Unigene_263538	942	Glucuronosyltransferase (*Zootermopsis nevdensis*)	6.257	1.077	-2.54	0.000105	0.0444
Unigene_252632	306	Acylphosphatase (*Acyrthosiphon pisum*)	57.208	6.835	-3.07	1.89E-05	0.0123
Unigene_224486	1527	Catalase (*Riptortus pedestris*)	21.707	8.362	-2.69	1.70E-05	0.0114

*E*.*sophia* infection influenced carbohydrate, lipid, and energy metabolism in the host. Some studies have found that trehalose content changed after parasitization [78,79]. In two treatment groups, maltose was degraded to glucose under the action of maltase. Beside the upregulation of maltase, other genes, in the citrate cycle and glycolysis, were over-expressed at 72AP, such as citrate synthase, aconitate hydratase, and glyceraldehydes 3-phosphate dehydrogenase. Glycolysis and the citrate cycle are carbohydrate metabolisms to produce ATP. Citrate synthase, aconitate hydratase and succinyl-CoA synthetase are three essential enzymes in the TCA cycle. Up-regulation of genes that control the synthesis of these enzymes showed that total ATP decreased in the organism. Therefore, the insect needed to obtain more energy by increasing the reaction rate of the TCA cycle. In addition, we found that the transcriptional level of cytochrome c oxidase and f-type H^+^-transporting ATPase were significantly enhanced. Cytochrome c oxidase is involved in ATP synthesis as a terminase of the mitochondrial inner membrane respiratory chain [80]. However, whether it can be regarded as evidence of enhanced respiration is not clear. Measurement of respiration rate should be investigated in future studies.

There are three types of ion transporting ATPases: P-type, V-type, and F-type. In organisms, their main function is to synthesize ATP and transport H^+^ [81]. There were more over-expressed f-type H^+^-ATPases at 72AP than at 24AP. This suggests that *E*. *sophia* parasitization of *B*. *tabaci* involved increased energy consumption. The host was regulated to produce more energy to supply to the parasitoid. Visser *et al*. found most wasps lacked a lipid synthesis mechanism and could not accumulate energy [82]. Therefore, it is reasonable to assume that the parasitoid may continually obtain energy from the host in order to complete its development.

## Conclusions

In summary, our study first presented comprehensive transcriptome profiles of *B*. *tabaci* in response to *E*. *sophia* parasitization using RNAseq. The most of differentially expressed genes of *B*. *tabaci* after parasization have potential roles in immunity, development and metabolism to meet parasitoids needs. The transcriptome profiles provided a basis for future research in elucidate the host-parasitoid interaction. In addition, the identified immune-, development and detoxification–related genes may be target for *B*. *tabaci* control.

## References

[1] Oliveira M, Henneberry T, Anderson P (2001) History, current status, and collaborative research projects for *Bemisia tabaci*. Crop Prot 20: 709–723.

[2] Li ZX (2006) Molecular phylogenetic analysis reveals at least five genetic races of *Bemisia tabaci* in China. Phytoparasitica 34:431–440.

[3] Costa H, Brown J (1991) Variation in biological characteristics and esterase patterns among populations of *Bemisia tabaci*, and the association of one population with silverleaf symptom induction. Entomologia experimentalis et applicata 61:211–219.

[4] Horowitz AR, Kontsedalov S, Khasdan V, Ishaaya I (2005) Biotypes B and Q of *Bemisia tabaci* and their relevance to neonicotinoid and pyriproxyfen resistance. Arch Insect Biochem Physiol 58:216–225. 15756703 10.1002/arch.20044

[5] Wan F, Zhang G, Liu S, Luo C, Chu D, Zhang Y, et al (2009) Invasive mechanism and management strategy of *Bemisia tabaci* (Gennadius) biotype B: Progress report of 973 Program on invasive alien species in China. Science in China Series C: Life Sciences 52:88–95. 10.1007/s11427-008-0135-4 19152088

[6] Hoddle M, Van Driesche R, Sanderson J (1998) Biology and use of the whitefly parasitoid *Encarsia formosa*. Annu Rev Entomol 43:645–669. 15012401 10.1146/annurev.ento.43.1.645

[7] Xiao Y, Chen J, Cantliffe D, Mckenzie C, Houben K, Osborne LS (2011) Establishment of papaya banker plant system for parasitoid, *Encarsia sophia* (Hymenoptera: Aphilidae) against *Bemisia tabaci* (Hemiptera: Aleyrodidae) in greenhouse tomato production. Biol Control 58:239–247.

[8] Jaworski CC, Chailleux A, Bearez P, Desneux N (2015) Apparent competition between major pest reduces pest population densities on tomato crop, but not yield loss. J Pest Sci 88: 793–803.

[9] Zhang YB, Yang NW, Sun LY, Wan FH (2015) Host instar suitability in two invasive whiteflies for the naturally occurring parasitoid *Eretmocerus hayati* in China. J Pest Sci 88: 225–234.

[10] Lin KJ, Lu YH, Wan P, Yang YZ, Wyckhuys KAG, Wu KM (2015) Simultaneous reduction in incidence of *Bemisia tabaci* (Hemiptera: Aleyrodidae) and *Sylepta derogate* (Lepidoptera:Pyralidae) using velvetleaf, *Abutilon theophrasti* as a trap crop. J Pest Sci. 88:49–56.

[11] Cruz PL, Baldin ELL, de Castro MJP (2014) Characterization of antibiosis to the silverleaf whitefly *Bemisia tabaci* biotype B (Hemiptera: Aleyrodidae) in cowpea entries. J Pest Sci. 87:639–645

[12] Moreno-Ripoll R, Gabarra R, Symondson WOC, King RA, Agusti N (2014) Do the interations among natural enemies compromise the biological control of the whitefly *Bemisia tabaci*? J Pest Sci 87:133–141.

[13] Cahill M, Gorman K, Day S, Denholm I (1996) Baseline determination and detection of resistance to imidacloprid in *Bemisia tabaci* (Homoptera: Aleyrodidae). Bull Entomol Res 86: 343–349.

[14] Horowitz AR, Kontsedalov S, Ishaaya I (2004) Dynamics of resistance to the neonicotinoids acetamiprid and thiamethoxamthiamethoxam in *Bemisia tabaci* (Homoptera: Aleyrodidae). J Econ Entomol 97: 2051–2056 15666764 10.1093/jee/97.6.2051

[15] Nauen R, Denholm I (2005) Resistance of insect pests to neonicotinoid insecticides: current status and future prospects. Arch Insect Biochem Physiol 58: 200–215. 15756698 10.1002/arch.20043

[16] Liang P, Tian YA, Biondi A, Desneux N, Gao XW (2012) Short-term and transgenerational effects of the neonicotinoid nitenpyram on susceptibility to insecticides in two whitefly species. Ecotoxicology 21:1889–1898. 10.1007/s10646-012-0922-3 22661314

[17] Turlings TC, Tumlinson JH, Lewis WJ (1990) Exploitation of herbivore-induced plant odors by host-seeking parasitic wasps. Science 250: 1251–1253. 17829213 10.1126/science.250.4985.1251

[18] Han P, Niu CY, Desneux N (2014) Identification of top-down forces regulating cotton aphid population growth in transgenic Bt cotton in centra China. PLoS ONE. 9:e102980 10.1371/journal.pone.0102980 25170907 PMC4149364

[19] Ali A, Desneux N, Lu YH, Liu B, Wu KM (2016) Characterization of the natural enemy community attacking cotton aphid in the Bt cotton ecosystem in northern China. Sci Rep 6: 24273 10.1038/srep24273 27075171 PMC4831012

[20] Askew RR, Shaw MR (1986) Parasitoid communities: their size, structure and development In: Waage J, Greathead D, editors. Insect Parasitoids. London: Academic pp. 225–263.

[21] Desneux N, Barta RJ, Hoelmer KA, Hopper KR, Heimpel GE (2009) Multifaceted determination of host specificity in an aphid parasitoid. Oecologia 160:387–398. 10.1007/s00442-009-1289-x 19219460

[22] Simmons AM, Abd-Rabou S (2005) Parasitism of *Bemisia tabaci* (Homoptera: Aleyrodidae) after multiple releases of *Encarsia sophia* (Hymenoptera: Aphelinidae) in three vegetable crops. J Agr Urban Entomol 22:73–77.

[23] Luo C, Liu TX (2011) Fitness of *Encarsia sophia* (Hymenoptera: Aphelinidae) parasitizing *Trialeurodes vaporariorum* and *Bemisia tabaci* (Hemiptera: Aleyrodidae). Insect Sci 18:84–91.

[24] Zang LS, Liu TX, Wan FH (2011) Reevaluation of the value of autoparasitoids in biological control. PLoS ONE 6: e20324 10.1371/journal.pone.0020324 21633501 PMC3102091

[25] Collier TR, Hunter MS (2001) Lethal interference competition in the whitefly parasitoids *Eretmocerus eremicus* and *Encarsia sophia*. Oecologia 129:147–154. 28547062 10.1007/s004420100706

[26] Mahadav A, Gerling D, Gottlieb Y, Czosnek H, Ghanim M (2008) Parasitization by the wasp *Eretmocerus mundus* induces transcription of genes related to immune response and symbiotic bacteria proliferation in the whitefly *Bemisia tabaci*. BMC Genomics 9. 10.1186/1471-2164-9-342 PMC2488360 18638407

[27] Chen XX, He JH, Shi ZH, Ma Y, Lou YG, Zhu ZR (2000) Research review of interaction between parasitoids and their host. *China Entomology of 21*^*th*^ *Century*——The Entomological Society of China academic annual conference paper highlights, 242–246.

[28] Zhang QQ, Wang F, Fang Q, Ye GY (2011) In vitro cellular response of Pieris rapae(Lepidoptera: Pieridae) hemocytes and the effects of *Pteromalus puparum* venom. Acta Entomologica Sinica 54: 1264–1273.

[29] Stettler P, Trenczek T, Wyler T, Pfister-Wilhelm R, Lanzrein B (1998) Overview of parasitism associated effects on host haemocytes in larval parasitoids and comparison with effects of the egg-larval parasitoid *Chelonus inanitus* on its host *Spodoptera littoralis*. J Insect Physiol 44:817–831. 12769877 10.1016/s0022-1910(98)00014-6

[30] Pennacchio F, Digilio M, Tremblay E (1995) Biochemical and metabolic alterations in *Acyrthosiphon pisum* parasitized by *Aphidius ervi*. Arch Insect Biochem Physiol 30: 351–367.

[31] Gan M, Miao XX, Ding DC (2003) Biochemical and metabolic alterations in *Aphis craccivora* Koch parasitized by *Lysiphlebus japonicas* Ashmead. Acta Phytophylacica Sinica 30: 255–260.

[32] Crozatier M, Ubeda J-M, Vincent A, Meister M (2004) Cellular immune response to parasitization in *Drosophila* requires the EBF orthologue collier. PLoS Biol 2: e196 15314643 10.1371/journal.pbio.0020196 PMC509289

[33] Williams MJ, Ando I, Hultmark D (2005) *Drosophila melanogaster* Rac2 is necessary for a proper cellular immune response. Genes to Cells 10: 813–823. 16098145 10.1111/j.1365-2443.2005.00883.x

[34] Ozsolak F, Milos PM (2011) RNA sequencing: advances, challenges and opportunities. Nat Rev Genet 12: 87–98. 10.1038/nrg2934 21191423 PMC3031867

[35] Qiu P, Benbow L, Liu SX, Greene JR, Wang LQ (2002) Analysis of a human brain transcriptome map. BMC Genomics 3:1–6. 11955288 10.1186/1471-2164-3-10 PMC103672

[36] Zhang F, Guo H, Zheng H, Zhou T, Zhou Y, Wang S, et al (2010) Massively parallel pyrosequencing-based transcriptome analyses of small brown planthopper (*Laodelphax striatellus*), a vector insect transmitting rice stripe virus (RSV). BMC Genomics 11:303 10.1186/1471-2164-11-303 20462456 PMC2885366

[37] Zeng V, Ewen-Campen B, Horch HW, Roth S, Mito T, Extavour CG (2013) Developmental gene discovery in a hemimetabolous insect: De novo assembly and annotation of a transcriptome for the *Cricket Gryllus* bimaculatus. PLoS ONE 8(5). 10.1371/journal.pone.0061479 PMC3646015 23671567

[38] Gandhe AS, Arunkumar KP, John SH, Nagaraju J (2006) Analysis of bacteria-challenged wild silkmoth, *Antheraea mylitta* (lepidoptera) transcriptome reveals potential immune genes. BMC Genomics 7: 1–10. 16857061 10.1186/1471-2164-7-184 PMC1559613

[39] Yang N, Xie W, Jones CM, Bass C, Jiao X, Yang X, et al (2013)Transcriptome profiling of the whitefly *Bemisia tabaci* reveals stage-specific gene expression signatures for thiamethoxam resistance. Insect Mol Biol 22: 485–496. 10.1111/imb.12038 23889345 PMC4229068

[40] Wertheim B, Kraaijeveld AR, Schuster E, Blanc E, Hopkins M, Pletcher SD, et al (2005) Genome-wide gene expression in response to parasitoid attack in *Drosophila*. Genome Biol 6: R94 16277749 10.1186/gb-2005-6-11-r94 PMC1297650

[41] Etebari K, Palfreyman RW, Schlipalius D, Nielsen LK, Glatz RV, Asgari S (2011) Deep sequencing-based transcriptome analysis of *Plutella xylostella* larvae parasitized by *Diadegma semiclausum*. BMC Genomics 12: 266–267. 21906285 10.1186/1471-2164-12-446 PMC3184118

[42] Wu SF, Sun FD, Qi YX, Yao Y, Fang Q, Huang J, et al (2013) Parasitization by *Cotesia chilonis* influences gene expression in fatbody and hemocytes of *Chilo suppressalis*. PLoS ONE 8: e74309 10.1371/journal.pone.0074309 24086331 PMC3781088

[43] Zhu JY, Yang P, Zhang Z, Wu GX, Yang B (2013) Transcriptomic immune response of *Tenebrio molitor* pupae to parasitization by *Scleroderma guani*. PLoS ONE 8: e54411 10.1371/journal.pone.0054411 23342153 PMC3544796

[44] Tang B, Chen J, Hou Y, Meng E (2014) Transcriptome immune analysis of the invasive beetle *Octodonta nipae* (Maulik) (Coleoptera: Chrysomelidae) parasitized by *Tetrastichus brontispae* Ferrière (Hymenoptera: Eulophidae). PLoS ONE 9:e91482 10.1371/journal.pone.0091482 24614330 PMC3948882

[45] Hoffmann JA (2003) The immune response of *Drosophila*. Nature 426:33–38. 14603309 10.1038/nature02021

[46] Chu D, Zhang YJ, Cong B, Xu BY, Wu QJ, Zhu GR (2005) Sequence analysis of mtDNA COI gene and molecular phylogeny of different geographical populations of *Bemisia tabaci* (Gennadius). Scientia Agricultura Sinica 38: 76–85.

[47] Grabherr MG, Haas BJ, Yassour M, Levin JZ, Thompson DA, Amit I, et al (2011) Fulllength transcriptome assembly from RNA-Seq data without a reference genome. Nat Biotechnol 29: 644–652. 10.1038/nbt.1883 21572440 PMC3571712

[48] Conesa A, Gotz S, Garcia-Gomez JM, Terol J, Talon M, Robles M (2005) Blast2GO: a universal tool for annotation, visualization and analysis in functional genomics research. Bioinformatics 21: 3674–3676. 16081474 10.1093/bioinformatics/bti610

[49] Ye J, Fang L, Zheng H, Zhang Y, Chen J, Zhang Z, et al (2006) WEGO: a web tool for plotting GO annotations. Nucleic Acids Res 34: W293–W297. 16845012 10.1093/nar/gkl031 PMC1538768

[50] Mortazavi A, Williams BA, McCue K, Schaeffer L, Wold B (2008) Mapping and quantifying mammalian transcriptomes by RNA-Seq. Nat Methods 5: 621–628. 10.1038/nmeth.1226 18516045 PMC13303166

[51] Hoffmann JA (2003) The immune response of *Drosophila*. Nature 426:33–38. 14603309 10.1038/nature02021

[52] Hoffmann JA (1995) Innate immunity of insects. Curr Opin Immunol 7: 4–10. 7772280 10.1016/0952-7915(95)80022-0

[53] Schmid-Hempel P (2005) Evolutionary ecology of insect immune defenses. Annu Rev Entomol 50: 529–551. 15471530 10.1146/annurev.ento.50.071803.130420

[54] Feldhaar H, Gross R (2008) Immune reactions of insects on bacterial pathogens and mutualists. Microbes Infect 10: 1082–1088. 10.1016/j.micinf.2008.07.010 18672091

[55] Nicolas E, Nappi AJ, Lemaitre B (1996) Expression of antimicrobial peptide genes after infection by parasitoid wasps in *Drosophila*. Dev Comp Immunol 20:175–181. 8955592 10.1016/0145-305x(96)00017-1

[56] Boulanger N, Lowenberger C, Volf P, Ursic R, Sigutova L, Sabatiern L, et al (2004) Characterization of a defensin from the sand fly *Phlebotomus duboscqi* induced by challenge with bacteria or the protozoan parasite Leishmania major. Infect Immun 72:7140–7146. 15557638 10.1128/IAI.72.12.7140-7146.2004 PMC529173

[57] Chiche L, Heitz A, Gelly J-C, Gracy J, Chau PT, Ha PT, et al (2004) Squash inhibitors: from structural motifs to macrocyclic knottins. Curr Protein Pept Sci 5:341–349. 15551519 10.2174/1389203043379477

[58] Christensen BM, Li J, Chen CC, Nappi AJ (2005): Melanization immune responses in mosquito vectors. Trends Parasitol 21:192–199. 15780842 10.1016/j.pt.2005.02.007

[59] Song KH, Jung MK, Eum JH, Hwang IC, Han SS (2008) Proteomic analysis of parasitized *Plutella xylostella* larvae plasma. J Insect Physiol 54:1271–1280. 10.1016/j.jinsphys.2008.06.010 18671979

[60] Bangi E, Pitsouli C, Rahme LG, Cagan R, Apidianakis Y (2012) Immune response to bacteria induces dissemination of Ras-activated *Drosophila* hindgut cells. Embo Rep 13:569–576. 10.1038/embor.2012.44 22498775 PMC3367237

[61] Corona M, Robinson G (2006) Genes of the antioxidant system of the honey bee: annotation and phylogeny. Insect mol biol 15:687–701. 17069640 10.1111/j.1365-2583.2006.00695.x PMC1847502

[62] Wang P, Oberley LW, Howe D, Jarvis DL, Chauhan G, Murhammer DW (2004) Effect of expression of manganese superoxide dismutase in baculovirus-infected insect cells. Appl Biochem Biotech 119:181–193. 10.1385/abab:119:2:181 15531788

[63] Zhu JY, Ze SZ, Stanley DW, Yang B (2014) Parasitization by *Scleroderma guani* influences expression of superoxide dismutase genes in *Tenebrio molitor*. Arch Insect Biochem Physiol 87:40–52. 10.1002/arch.21179 25042129

[64] Takeda T, Nakamatsu Y, Tanaka T (2006) Parasitization by *Cotesia plutellae* enhances detoxifying enzyme activity in *Plutella xylostella*. Pestic Biochem Physiol 86:15–22.

[65] Parsell D, Lindquist S (1993) The function of heat-shock proteins in stress tolerance: degradation and reactivation of damaged proteins. Annu Rev Genet 27:437–496. 8122909 10.1146/annurev.ge.27.120193.002253

[66] Qian M, Ren S, Hu Q (2005) Effects of endoparasitism by *Encarsia bimaculata* on the titers of juvenile hormones and 20—hydroxyecdysone in *Bemisia tabaci* nymphs. Acta entomologica Sinica 49:568–573.

[67] Hu JS, Gelman DB, Blackburn MB (2002) Growth and development of *Encarsia formosa* (Hymenoptera: Aphelinidae) in the greenhouse whitefly, *Trialeurodes vaporariorum* (Homoptera: Aleyrodidae): effect of host age. Arch Insect Biochem Physiol 49:125–136. 11857673 10.1002/arch.10015

[68] Zhu JY, Ye GY, Hu C (2008) Advance in the studies on molecular mechanisms of parasitoid manipulation of host. Acta Phytophylacica Sinica 35:563–568.

[69] Rath S, Sinha B (2005) Parasitization of fifth instar tasar silkworm, *Antheraea mylitta*, by the uzi fly, *Blepharipa zebina*; a host–parasitoid interaction and its effect on host’s nutritional parameters and parasitoid development. J Invertebr Pathol 88:70–78. 15707871 10.1016/j.jip.2004.09.006

[70] Kaeslin M, Pfister-Wilhelm R, Lanzrein B (2005) Influence of the parasitoid *Chelonus inanitus* and its polydnavirus on host nutritional physiology and implications for parasitoid development. J Insect Physiol 51:1330–1339. 16203013 10.1016/j.jinsphys.2005.08.003

[71] Gilbert LI, Granger NA, Roe RM (2000) The juvenile hormones: historical facts and speculations on future research directions. Insect Biochem Mol Biol 30:617–644. 10876106 10.1016/s0965-1748(00)00034-5

[72] Anspaugh DD, Roe RM (2005) Regulation of JH epoxide hydrolase versus JH esterase activity in the cabbage looper, *Trichoplusia ni*, by juvenile hormone and xenobiotics. J Insect Physiol 51:523–535. 15893999 10.1016/j.jinsphys.2004.12.008

[73] Ntambi JM, Miyazaki M, Dobrzyn A (2004) Regulation of stearoyl-CoA desaturase expression. Lipids 39:1061–1065. 15726820 10.1007/s11745-004-1331-2

[74] Zhang R, Zhang YH, Shao D, Wang LD, Gong DQ (2013) The function and regulation of stearoyl-CoA desaturase gene. Chinese Bulletin of Life Sciences 4: 008.

[75] Zhang SZ, Wan FH, Zhang F, Hua BZ (2003) Parasitic suitability of two strains of *Encarsia formosa* on *Bemisia tabaci*. Chinese J Biol Control 19: 149–153.

[76] Alloatti A, Gupta S, Gualdrón-López M, Nguewa PA, Altabe SG, Deumer G, et al (2011) Stearoyl-CoA desaturase is an essential enzyme for the parasitic protist *Trypanosoma brucei*. Biochem Bioph Res Co 412:286–290. 10.1016/j.bbrc.2011.07.084 21820408

[77] Holt HL, Aronstein KA, Grozinger CM (2013) Chronic parasitization by Nosema microsporidia causes global expression changes in core nutritional, metabolic and behavioral pathways in honey bee workers (*Apis mellifera*). BMC Genomics 14:799 10.1186/1471-2164-14-799 24245482 PMC4046765

[78] Dahlman DL, Vinson SB (1977) Effect of calyx fluid from an insect parasitoid on host hemolymph dry weight and trehalose content. J Invertebr Pathol 29:227–229.

[79] Nakamatsu Y, Tanaka T (2004) Correlation between concentration of hemolymph nutrients and amount of fat body consumed in lightly and heavily parasitized hosts (*Pseudaletia separata*). J Insect Physiol 50:135–141. 15019514 10.1016/j.jinsphys.2003.10.005

[80] Li LZ, Huang ZX (2001) Progresses in Cytochrome c Oxidase Studies. Chinese J Inorg Chem 17: 761–774.

[81] Song LX, Wang RX (1989) Structure and function on ion transporting ATPase. Prog Physiol Sci 20: 334–338. 2483758

[82] Visser B, Le Lann C, den Blanken FJ, Harvey JA, van Alphen JJ, Ellers J (2010) Loss of lipid synthesis as an evolutionary consequence of a parasitic lifestyle. PNAS 107: 8677–8682. 10.1073/pnas.1001744107 20421492 PMC2889307

